# Age-Dependent Changes in Geometry, Tissue Composition and Mechanical Properties of Fetal to Adult Cryopreserved Human Heart Valves

**DOI:** 10.1371/journal.pone.0149020

**Published:** 2016-02-11

**Authors:** Daphne van Geemen, Ana L. F. Soares, Pim J. A. Oomen, Anita Driessen-Mol, Marloes W. J. T. Janssen-van den Broek, Antoon J. van den Bogaerdt, Ad J. J. C. Bogers, Marie-José T. H. Goumans, Frank P. T. Baaijens, Carlijn V. C. Bouten

**Affiliations:** 1 Soft Tissue Biomechanics & Engineering, Department of Biomedical Engineering, Eindhoven University of Technology, Eindhoven, Netherlands; 2 Institute for Complex Molecular Systems, Eindhoven University of Technology, Eindhoven, Netherlands; 3 Heart Valve Bank Rotterdam, Department of Cardio-Thoracic Surgery, Erasmus University Medical Center, Rotterdam, Netherlands; 4 Department of Cardio-Thoracic Surgery, Erasmus University Medical Center, Rotterdam, Netherlands; 5 Department of Molecular Cell Biology, Leiden University Medical Center, Leiden, Netherlands; University of California, San Diego, UNITED STATES

## Abstract

There is limited information about age-specific structural and functional properties of human heart valves, while this information is key to the development and evaluation of living valve replacements for pediatric and adolescent patients. Here, we present an extended data set of structure-function properties of cryopreserved human pulmonary and aortic heart valves, providing age-specific information for living valve replacements. Tissue composition, morphology, mechanical properties, and maturation of leaflets from 16 pairs of structurally unaffected aortic and pulmonary valves of human donors (fetal-53 years) were analyzed. Interestingly, no major differences were observed between the aortic and pulmonary valves. Valve annulus and leaflet dimensions increase throughout life. The typical three-layered leaflet structure is present before birth, but becomes more distinct with age. After birth, cell numbers decrease rapidly, while remaining cells obtain a quiescent phenotype and reside in the ventricularis and spongiosa. With age and maturation–but more pronounced in aortic valves–the matrix shows an increasing amount of collagen and collagen cross-links and a reduction in glycosaminoglycans. These matrix changes correlate with increasing leaflet stiffness with age. Our data provide a new and comprehensive overview of the changes of structure-function properties of fetal to adult human semilunar heart valves that can be used to evaluate and optimize future therapies, such as tissue engineering of heart valves. Changing hemodynamic conditions with age can explain initial changes in matrix composition and consequent mechanical properties, but cannot explain the ongoing changes in valve dimensions and matrix composition at older age.

## Introduction

End stage valvular disease is commonly treated with heart valve replacement to alleviate cardiac, pulmonary, or systemic problems due to the disease. Semilunar valves, in particular the aortic valve, are most often replaced. Although current heart valve alternatives enhance survival and quality-of-life of most patients, they have several limitations. The most important drawback is that they do not consist of living tissue and, therefore, do not grow, repair, and remodel after implantation. Especially for pediatric and adolescent patients, who require multiple valve replacements, this is a significant problem. In this regard replacement of the diseased aortic valve by the autologous pulmonary valve autograft (Ross procedure), common for pediatric and adolescent patients, is considered clinically effective [1]. Nevertheless, redo surgery for autograft failure in second postoperative decade is not uncommon and the replacement pulmonary valve is at risk for an additional operation or intervention as well.

Heart valve tissue engineering (HVTE) seeks to overcome the current limitations of valve prostheses, allografts and autografts, by creating a living heart valve replacement that can grow and adapt in response to changing functional demands. Key design parameters for HVTE are: valve geometry and morphology, cell type, extracellular matrix (ECM) composition and architecture, tissue mechanical properties, and growth and remodeling potential, which may all change with the target age group for valve replacement. As the properties of native human heart valves represent the ideal blueprint for HVTE [2], data from human valves of different ages should be used for optimizing HVTE for different target groups.

So far, valvulogenesis and tissue morphogenesis of heart valves have been mainly studied using valves of animal origin (e.g. porcine) [2–7]. Studies with human valves mostly concentrate on fetal or adult valves [8–14], while studies on human pediatric and adolescent valves are only sparsely available [10,15,16]. Hence, the development of structure-function properties from young to old age is largely unknown. In addition, there is no consensus on potential differences in structure-function properties between human pulmonary and aortic valves.

Here, we provide a comprehensive data set on aortic and pulmonary valve properties at different stages of growth and development (fetal, child, adolescent, and adult) that can be used for the development, application, and evaluation of living, age-specific heart valve replacements. The development of structure-function properties and valve remodeling was assessed from quantitative (dimensions, biochemical assays, mechanical testing) and qualitative (histology) measures of the geometry, morphology, composition, and mechanical properties of sixteen pairs of structurally unaffected human aortic and pulmonary heart valves.

## Methods

### Tissue preparation

Sixteen sets of cryopreserved healthy human aortic and pulmonary valves from the same donor (0–53 year, n = 32 valves) were obtained with written informed consent from Dutch postmortem donors. For the pediatric valves, the written informed consent was obtained from the next of kin or legal guardians. All postnatal valves were assessed to be unfit for implantation and were obtained from, and after approval for the current study by, the Heart Valve Bank Rotterdam (Erasmus University Medical Center, Rotterdam, The Netherlands). The fetal valves (1 set; 21 weeks of gestation) were obtained after elective abortion following individual written informed consent, after approval of the current study by the medical ethical committee of the Leiden University Medical Center (MEC-P08.087). The investigation of the fetal as well as the postnatal valves was conform the principles outlined in the Declaration of Helsinki. All valves were structurally and mechanically unaffected as assessed by macroscopic observations at time of cryopreservation at the Heart Valve Bank and after thawing by the researchers. The cause of death of the donors was not related to valvular disease or conditions known to precede valvular disease. Earlier studies demonstrated that the applied cryopreservation and thawing protocol applied to these valves did not affect the structural integrity of collagen and elastin [17] and mechanical properties [18]. The fetal valves were only studied with histology due to the size of these valves.

**Fig 1:**
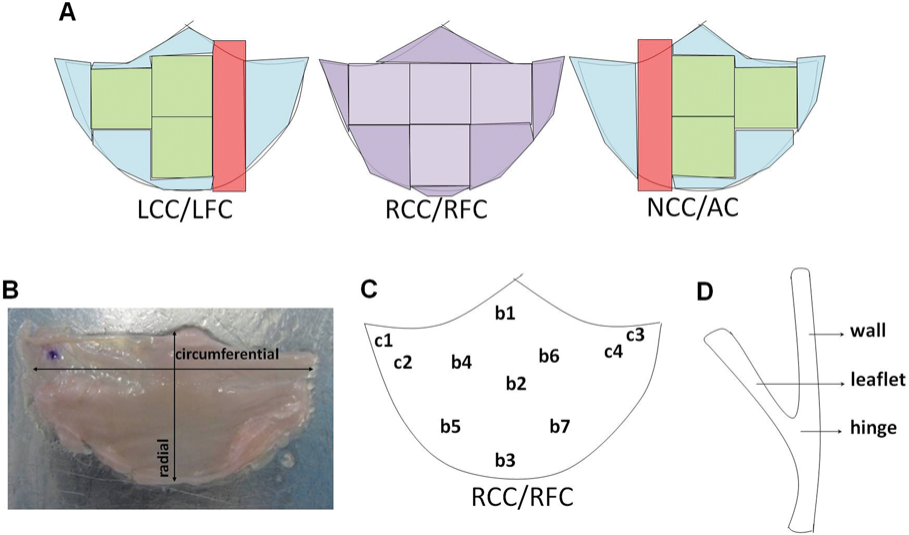
Schematic overview of the leaflet sectioning and assignment to the different experiments. (A) Schematic overview of sectioning of one heart valve for analyses. Samples for biaxial tensile testing are indicated in green; parts for histology in red. The right coronary cusp (RCC) and right facing cusp (RFC) were used for indentation tests (purple). Afterwards, this leaflet was used for biaxial tensile testing. Leftover tissue (blue) was freeze-dried for biochemical assays. LCC: Left coronary cusp; LFC: Left facing cusp; NCC: non-coronary cusp; AC: anterior cusp. (B) The size of the leaflets was measured in circumferential and radial direction (arrows). (C) Indentation tests were performed in the commissural (c1 –c4) and belly (b1 –b7) region of the RCC/RFC. (D) Schematic cross-section of the postnatal heart valve used for histology, depicting the wall, leaflet and hinge regions.

The cryopreserved valves were stored at -80°C and thawed just prior to ECM analysis and mechanical testing. The thawing protocol was similar for all studies heart valves. The thawed valves were cut according to a cutting scheme in preparation for the pre-defined analyses (Fig 1 and S1 Table). Samples for histology and biochemical assays were respectively fixed in formalin or snap-frozen within 24 hours, while mechanical testing was performed within 48 hours after thawing.

### Valve dimensions and morphology

Valve dimensions were quantified by defining the annulus diameter, leaflet size (both radial and circumferential), and leaflet thickness. The annulus diameter and gross morphological appearance (e.g. presence of fibrosis or artheroma) of the valve leaflets were assessed upon explantation by the Heart Valve Bank Rotterdam. The size of the right (coronary) leaflet was measured after excision in circumferential and radial direction to assess the dimensions of the leaflets (S1B Fig). The thickness of the leaflets was measured as part of the indentation tests (see below).

### Cell phenotype, tissue composition and maturation

Pieces of the leaflets (leaflet, hinge region and part of the wall; Fig 1D) were fixed overnight in formalin, processed and subsequently embedded in paraffin. They were sectioned at 10 μm thickness to qualitatively study phenotype of the valvular interstitial cells and matrix composition using histology (H&E: tissue morphology, Masson Trichrome: collagen, Verhoeff-van Gieson: collagen and elastin, Safranin-O: proteoglycans) and immunofluorescent stainings (elastin, collagen type I and III, and alpha smooth muscle actin (αSMA)). The leaflet parts for biochemical assays (2–4 tests per valve) were lyophilized and digested in papain solution (100 mM phosphate buffer [pH = 6.5], 5 mM L-cystein, 5 mM EDTA, and 125–140 μg papain/ml) to determine total cell number (DNA content, μg/mg dry weight), matrix composition (sulfated glycosaminoglycans (sGAG) and hydroxyproline content, μg/mg dry weight), and collagen maturation (# cross-links per collagen triple helix). For details on the qualitative and quantitative measures, see S1 Appendix.

### Indentation tests

To characterize local tissue mechanical properties, indentation tests were performed on all sets (n = 30 valves) in the belly region (~7 indentations per leaflet) and near the commissures (~4 indentations per leaflet) of hydrated right (coronary) leaflets (Fig 1C). For indentation tests, the fibrosa-side of the leaflet was placed face-down on a glass (coverslip) and the tests were performed at room temperature as described previously [19]. Briefly, a spherical sapphire indenter (diameter 2 mm) was used to compress the tissue with a constant indentation speed of 0.01 mm/s. At the indented locations, one preconditioning cycle followed by two indentation tests were performed to determine leaflet stiffness (E-modulus), which was calculated from the force-indentation curve up to 20% indentation. When the indenter touches the leaflet, a drop in the force signal was observed. The height of the indenter with respect to the coverslip at that moment was taken as the thickness of the indented sample.

### Biaxial tensile tests

To further analyze mechanical properties of the valves, biaxial tensile tests were performed on 1 to 3 sets of valve leaflets per age group, except for the fetal valves (n = 12 valves in total, see S1 Table) as previously described [20]. In brief, rectangular samples were cut (see Fig 1), kept hydrated and placed on aluminum foil to mount the samples in a BioTester 5000 device (CellScale, Waterloo, Canada) using a 5N load cell and a BioRakes mounting system with 0.7 mm space between the pins. The samples were then tested while submersed in PBS at room temperature. The samples were biaxially stretched to peak values (failure) in circumferential (ε_c_) and radial (ε_r_) leaflet direction at a strain rate of the initial sample length per minute and following a step-wise protocol. A first series of strains was applied, according to ε_c_: ε_r_ (% strain) = 0:60; 11.5:55; 23: 11.5: 25:0 to capture the non-linear mechanical behavior of the leaflets. The samples were unloaded and left to recover for 1 minute in between steps. Next, the strains were sequentially increased during up to 5 additional series in steps of 5% or 10% until failure. Before each series, the samples were preconditioned for 10 cycles first to the max ε_c_ and then to the max ε_r_ of the group.

The final stress-strain curves, where stresses were normalized for tissue thickness (Cauchy stress), were averaged per tested valve (max. 6 samples per valve, see Fig 1) and plotted for both radial and circumferential direction. The average curves stop when the first sample failed.

In order to achieve easily interpretable figures for mechanical properties and to account for inter-subject variation in non-linear stress-strain behavior, we defined stiffness values (E-modulus) from the tangent of the fitted slope of the linear strain-hardening region of the stress-strain curves and tissue extensibility from the x-intercept of the slope of the strain-hardening part of the stress-strain curves. These values were averaged per tested valve using the individual stress-strain curves that reached the strain-hardening phase.

### Data analyses and mapping hemodynamic function

Data are presented as mean ± the standard error of the mean. Postnatal valves were sub-divided into three age groups based on the age of the available donor valves (S1 Table): child (2 months to 11 years; n = 5), adolescent (18–22 years; n = 4), and adult (38–53 years; n = 6). Fetal valves were not included in statistical analyses. Relationships between valve properties or between valve properties and age were identified with correlation analysis (with r the correlation coefficient). Statistical differences (p<0.05) between age groups and the aortic and pulmonary valves were tested with two-way ANOVA, followed by Bonferroni post-hoc testing. GraphPad Prism software (GraphPad Software, Inc, USA) was used for the analyses.

In an attempt to correlate valve geometry and tissue composition with changing postnatal hemodynamic conditions, we collected hemodynamic data from several databases, including The National Heart Lung and Blood Institue, MedScape and literature [21–23].

## Results

### Dimensions of human heart valve leaflets continue to increase with age

**Fig 2:**
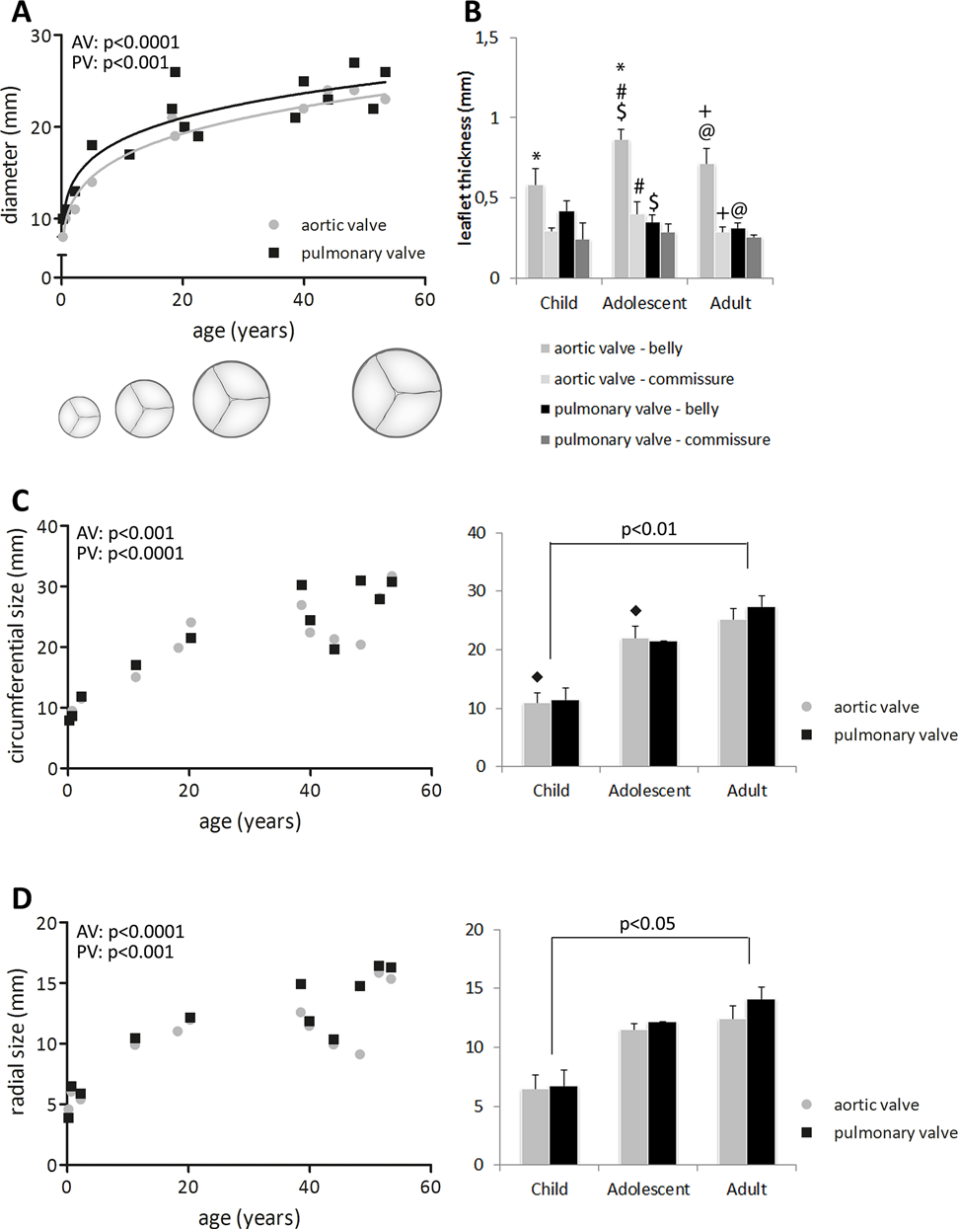
Evolution of valve dimensions. (A) Increase in annulus diameter in the aortic (grey) and pulmonary (black) valve. The diameter increases rapidly early in life and slowly, but continuously, thereafter. The pulmonary valve is slightly larger compared to the aortic valve. (B) The thickness of the leaflets is heterogenous and larger in the belly compared to the commissures, which is similar for all age groups. Aortic valves are slightly thicker than pulmonary valves, especially in the belly. (C+D) Changes in leaflet geometry (left side: all data points for correlation analysis with age; right side: grouped data). The leaflet size measured in circumferential (C) and radial (D) direction increases with age. Significant differences between groups (p<0.05) are indicated by paired symbols. AV: aortic valve, PV: pulmonary valve.

The annulus diameter of the heart valves increased with age (p<0.001; Fig 2A), with the pulmonary annulus slightly larger than the aortic annulus. As expected, annulus size increased rapidly in the pediatric group and slowly thereafter, with continued, but slow increase up to old age. Concomitantly, leaflet size increased in circumferential and radial direction with age, and this increase was similar for both valves (p<0.001; Fig 2C and 2D).

The thickness of the aortic valve leaflets was heterogeneous (Fig 2B). Overall, the aortic valve leaflets were thicker than the pulmonary valve leaflets. This difference was predominant in the belly of adolescent and adult valves (p<0.001). The commissural regions of the aortic leaflets were thinner than the belly region, but only significant in the adolescent and adult valves (p<0.001).

In the pediatric valves, fibrosis was not observed at all. In the valves from the adolescent group mild fibrosis was seen, though not in all valves. However, adult valves all showed some degree of fibrosis. Fibrosis was mostly found in the central belly region, and incidentally at the fixed edge of the leaflet. Some adult valves showed atheroma spots at the fixed edge of the leaflet.

### Three-layered structure is present before birth, but becomes more distinct with age

**Fig 3:**
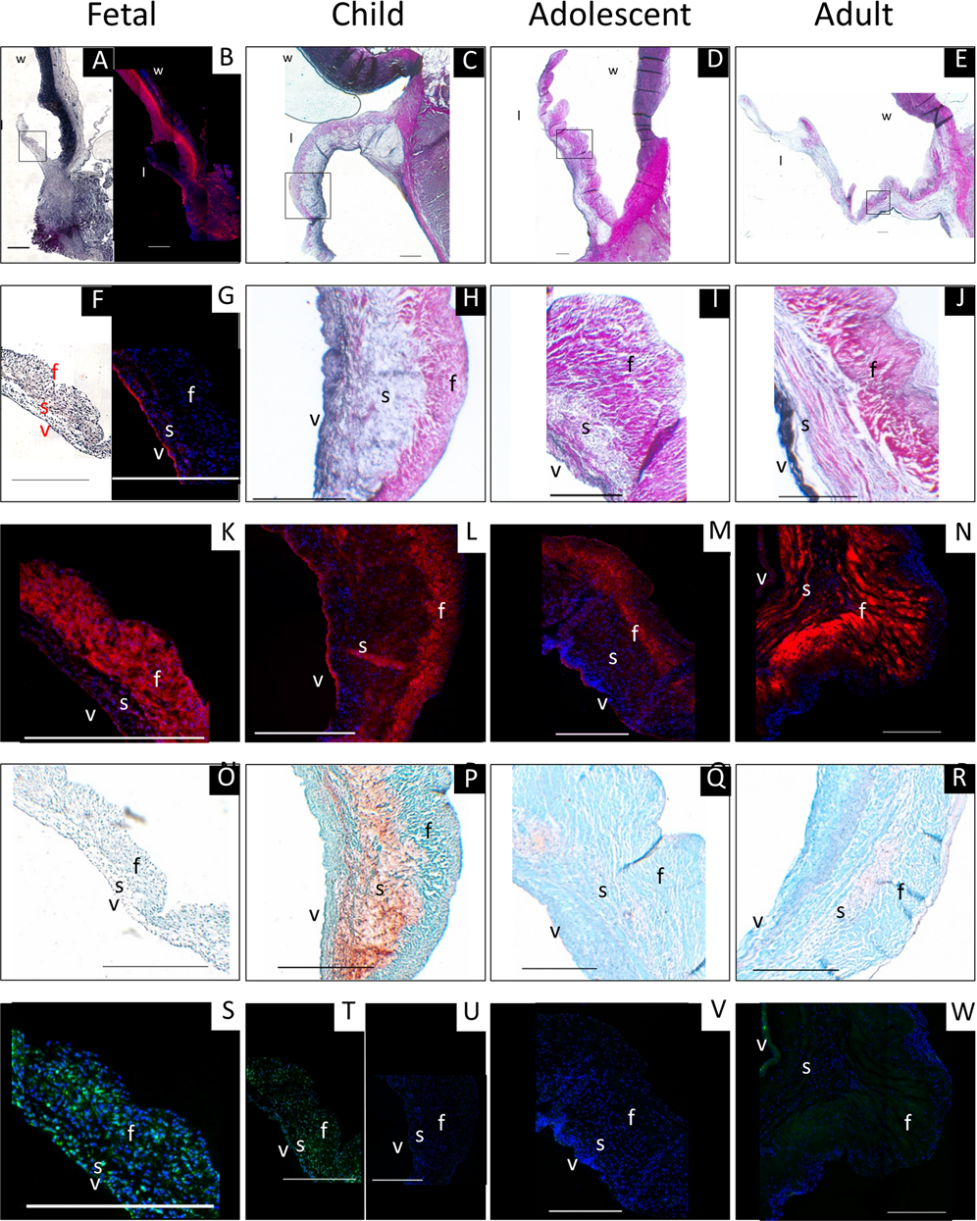
Representative histological and immunofluorescent stainings on aortic valves. Figures A-E represent the whole aortic valve (leaflet, hinge region, and wall, while in E-W a representative part of the leaflet is shown. (A, C-F, H-J) Verhoeff-Van Gieson staining for collagen (red) and elastin (black). (B, G) Elastin was observed in the fetal valve using immunofluorescence (red). (K-N) Collagen type I (red) was predominant in the fibrosa. (O-R) Safranin-O staining showed proteoglycan presence (red/orange) mainly in the spongiosa and the hinge region. (S-W) αSMA (immunofluorescence; green) with cell nuclei (in blue). In the leaflets of the fetal (S) and 8-month old donor (T) αSMA-positive cells were observed, while in the older leaflets almost no αSMA-positive cells were observed (U-W). Scale bar: 500 μm. l: leaflet; w: wall; f: fibrosa; s: spongiosa; v: ventricularis.

The typical three-layered leaflet structure was clearly visible in all studied valves, even in the fetal valves (Fig 3), but became more pronounced with age. Collagen was predominantly found in the fibrosa, as demonstrated by the Masson Trichrome (not shown) and Verhoeff-Van Gieson staining (Fig 2A, 2C–2F and 2H–2J). Here, collagen was mainly characterized as type I (Fig 3K–3N), whereas collagen type III was present throughout the leaflet (data not shown). Elastin was observed in the ventricularis (Fig 3A–3J). In the fetal valve, elastin was clearly observed with the elastin immunofluorescent staining, but not with the Verhoeff-Van Gieson staining (Figs 3B versus 3A and 2G vs. 2F). Proteoglycans were observed in the spongiosa of the leaflets and in the hinge region of the valves (Fig 3O–3R). Since the differences between the aortic and pulmonary valves were minimal, the aortic valve was chosen to represent the observed findings (S1 Fig for the histology of the pulmonary valve).

### Cellular content and phenotype change rapidly after birth

**Fig 4:**
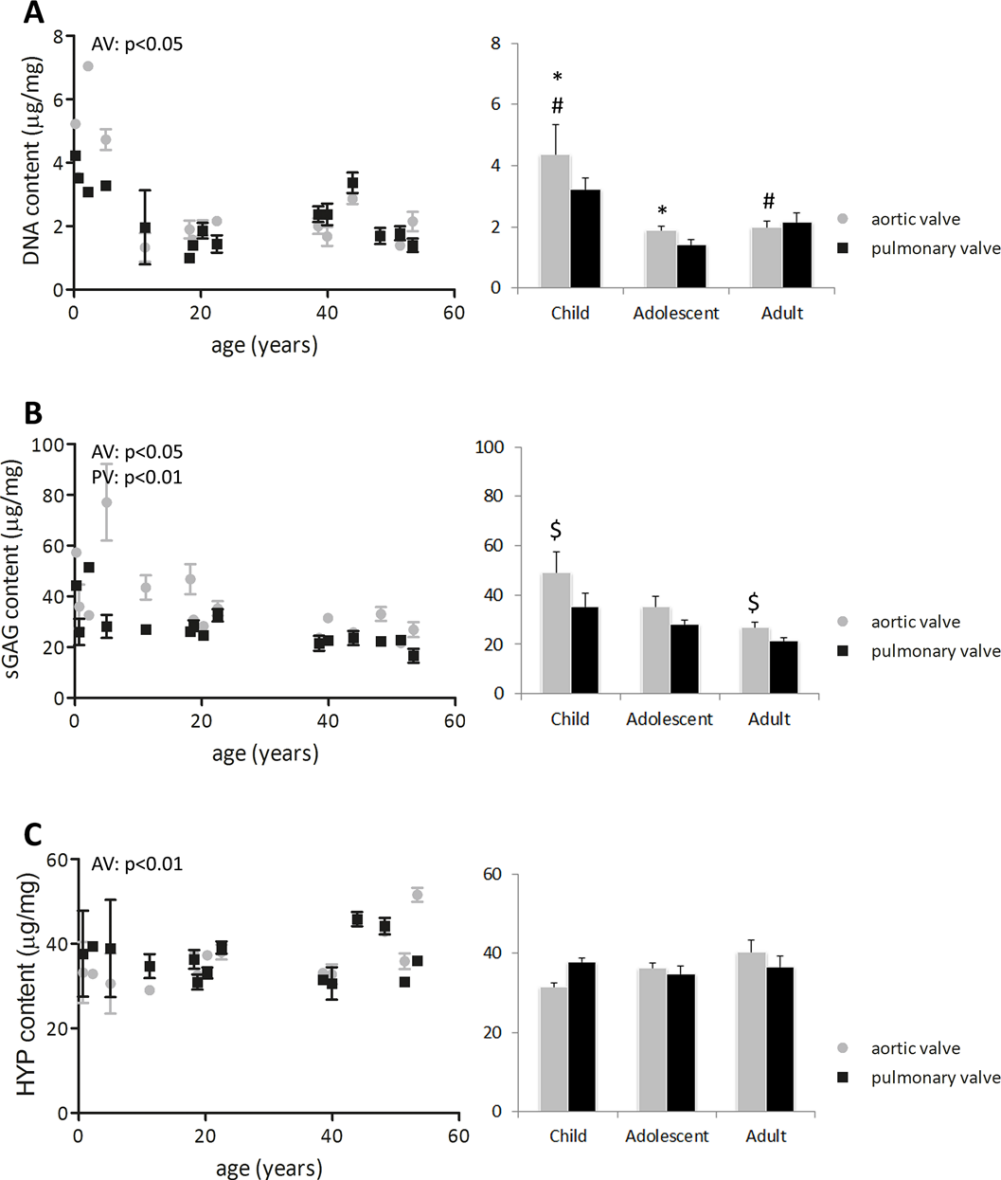
Changes in DNA-, sGAG-, and hydroxyproline (HYP) content. Changes in DNA- (A), sGAG- (B), and hydroxyproline (HYP) content (C) in μg/mg dry weight (left side: all data points used for correlation analysis with age; right side: grouped data). DNA content is higher in children, especially in the first years of life (0–4 years), compared to the adolescents and adults. sGAG content decreases with age. Hydroxyproline content increases with age in the aortic valve. Differences between groups (p<0.05) are indicated by paired symbols. AV: aortic valve, PV: pulmonary valve.

Histological analyses (Fig 3) suggest that cell content decreases with age. Total cell number (DNA content) was indeed higher in the pediatric aortic leaflets compared to the adolescent and adult aortic leaflets (p<0.01; Fig 4A). Especially, the leaflets from the youngest donors contained more DNA than the older leaflets. No differences in cell number were found between the aortic and pulmonary valve. Interestingly, in the fetal and pediatric leaflets, cells were observed throughout all layers of the leaflet, while the cells in the adolescent and adult leaflets were mainly located in the spongiosa and ventricularis.

αSMA-positive cells were frequently observed in the leaflets of the fetal and 8-month old donor, but were only sparsely observed in the older valve leaflets (≥ 4 years), indicating a more quiescent cell phenotype (Fig 3S–3W). When present, αSMA-positive cells were observed in the hinge region and the arterial wall for the fetal and pediatric valves, and predominantly in the arterial wall for the adult valves.

### Matrix composition shifts toward decreased sGAG, increased collagen content and maturity with age

The sGAG content decreased with age in both the aortic (p<0.05) and pulmonary valves (p<0.01; Fig 4B). The decrease in sGAG with age was most obvious for the aortic valve, especially from pediatric to adult aortic valves (p<0.01). In addition to a decrease in sGAGs, the hydroxyproline content, as a measure of valvular collagen, increased with age in the aortic valve (p<0.01; Fig 4C). The hydroxyproline-to-sGAG ratio increased with age for both valves (p<0.05; data not shown). Interestingly, pediatric aortic valves contained more sGAG relative to hydroxyproline, while in the adult aortic valves more hydroxyproline compared to sGAG was present.

**Fig 5:**
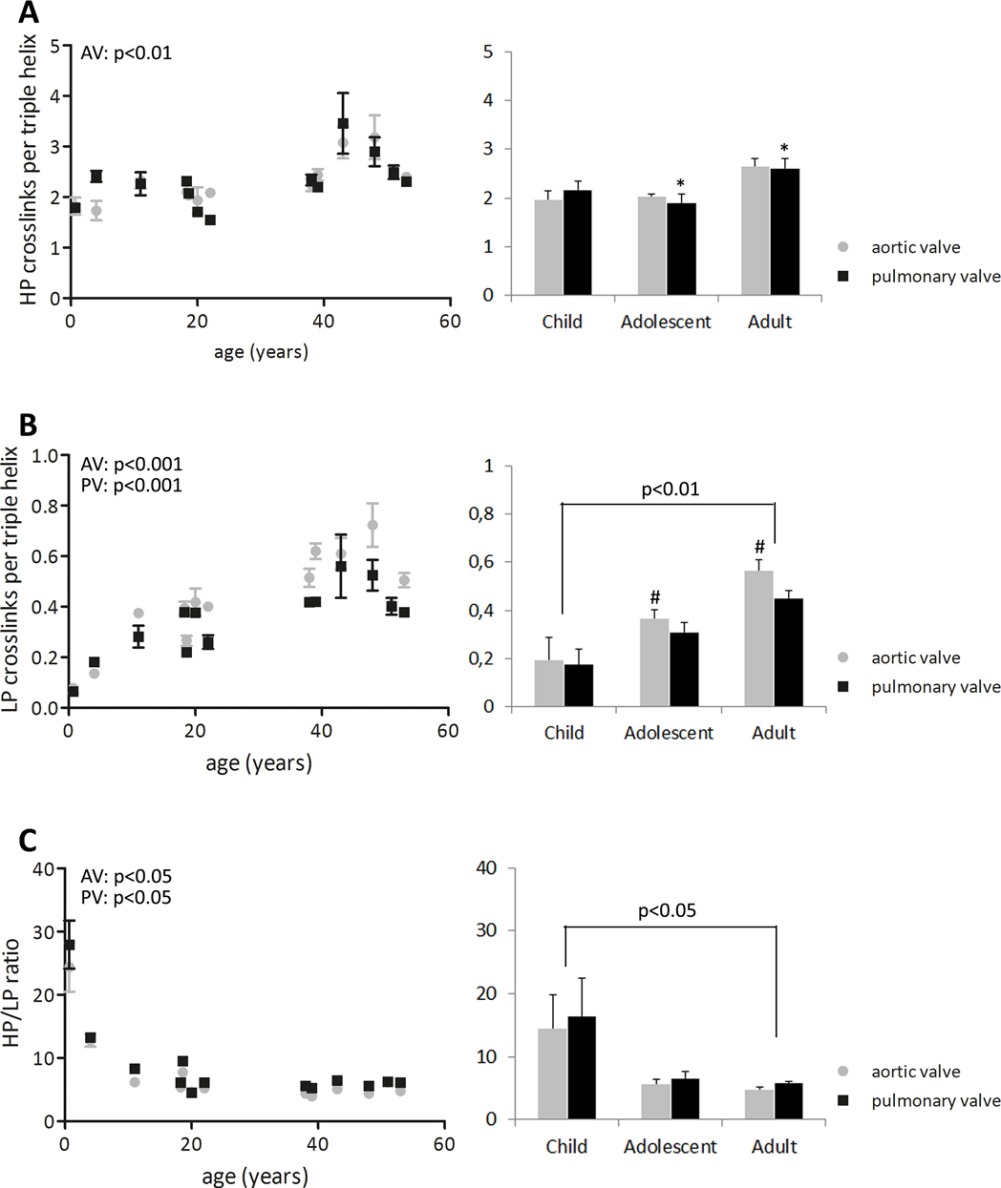
Changes in collagen HP, LP cross-links, and the HP-to-LP ratio. Changes in collagen HP (A), LP cross-links (B), and the HP-to-LP ratio (C) in μg/mg dry weight (left side: all data points used for correlation analysis with age; right side: grouped data). HP cross-links density increases with age in the aortic valve. In the pulmonary valve, the HP cross-link density increases from adolescent to adult age. LP cross-link density increases with age in both valves. Only in the adult valves, LP cross-link density is different between the aortic and pulmonary valve. In both valves, the HP-to-LP ratio decreases with age, but particularly between childhood and adolescence. Differences between groups (p<0.05) are indicated by paired symbols. AV: aortic valve, PV: pulmonary valve.

The collagen matrix of both aortic and pulmonary valves matured with age, as indicated by an increase in cross-link density (Fig 5). In the aortic valve, HP collagen cross-link density increased slightly throughout life (p<0.01), whereas in the pulmonary valve HP cross-link density only increased from adolescent to adult age (p<0.05; Fig 5A). LP cross-link density increased with age in both aortic and pulmonary valves (p<0.001; Fig 5B). HP cross-link density was always higher than LP cross-link density, but the HP-to-LP ratio decreased with age in both valves (p<0.05), predominantly from childhood to adolescence (p<0.05; Fig 5C).

### Leaflet stiffness and extensibility change with age

**Fig 6:**
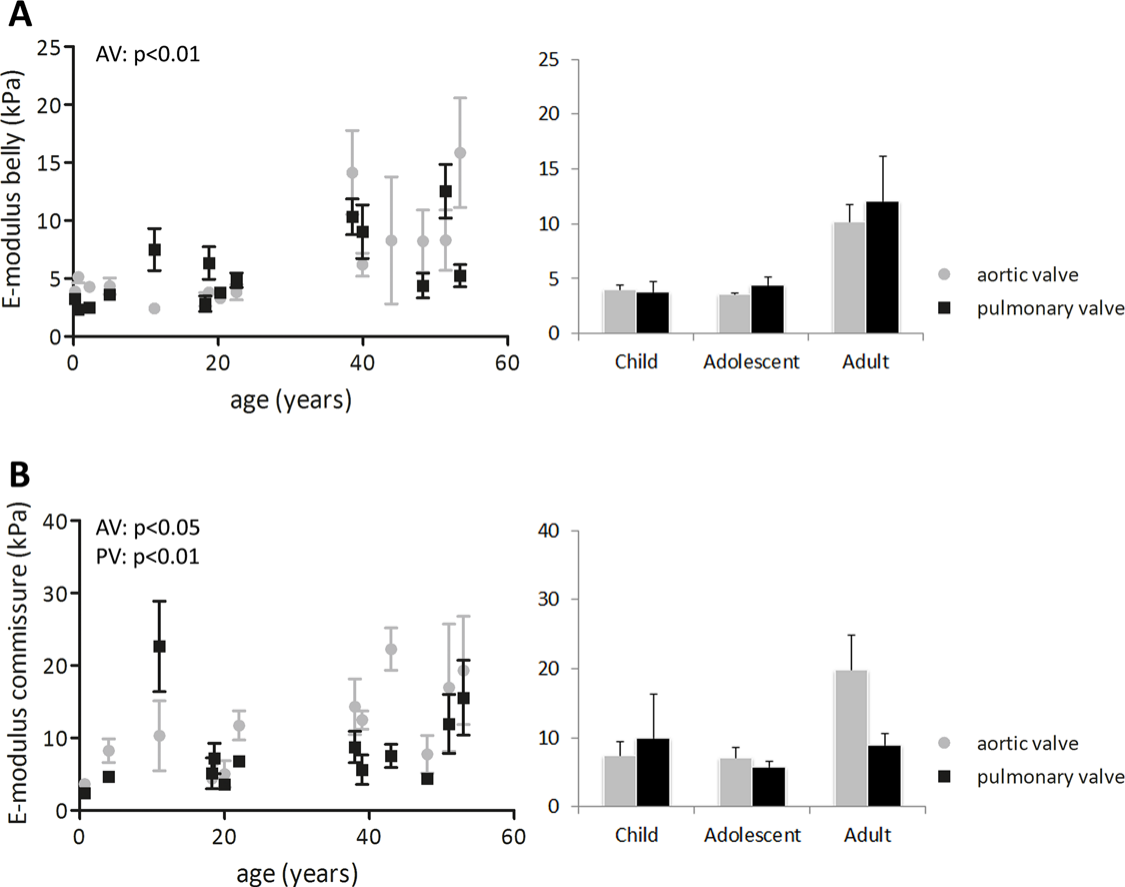
Changes in stiffness at low strains as measured with indentation tests. The stiffness was computed in the belly (A) and commisures (B) (left side: all data points used for correlation analysis with age; at the right side). In the aortic valve, the stiffness in the belly and commissures increase with age, whereas in the pulmonary valve only the commissural stiffness increases with age. In both valves, E-moduli show a steep increase from adolescence to adulthood. Significant differences between groups (p<0.05) are indicated by paired symbols. AV: aortic valve, PV: pulmonary valve.

**Fig 7:**
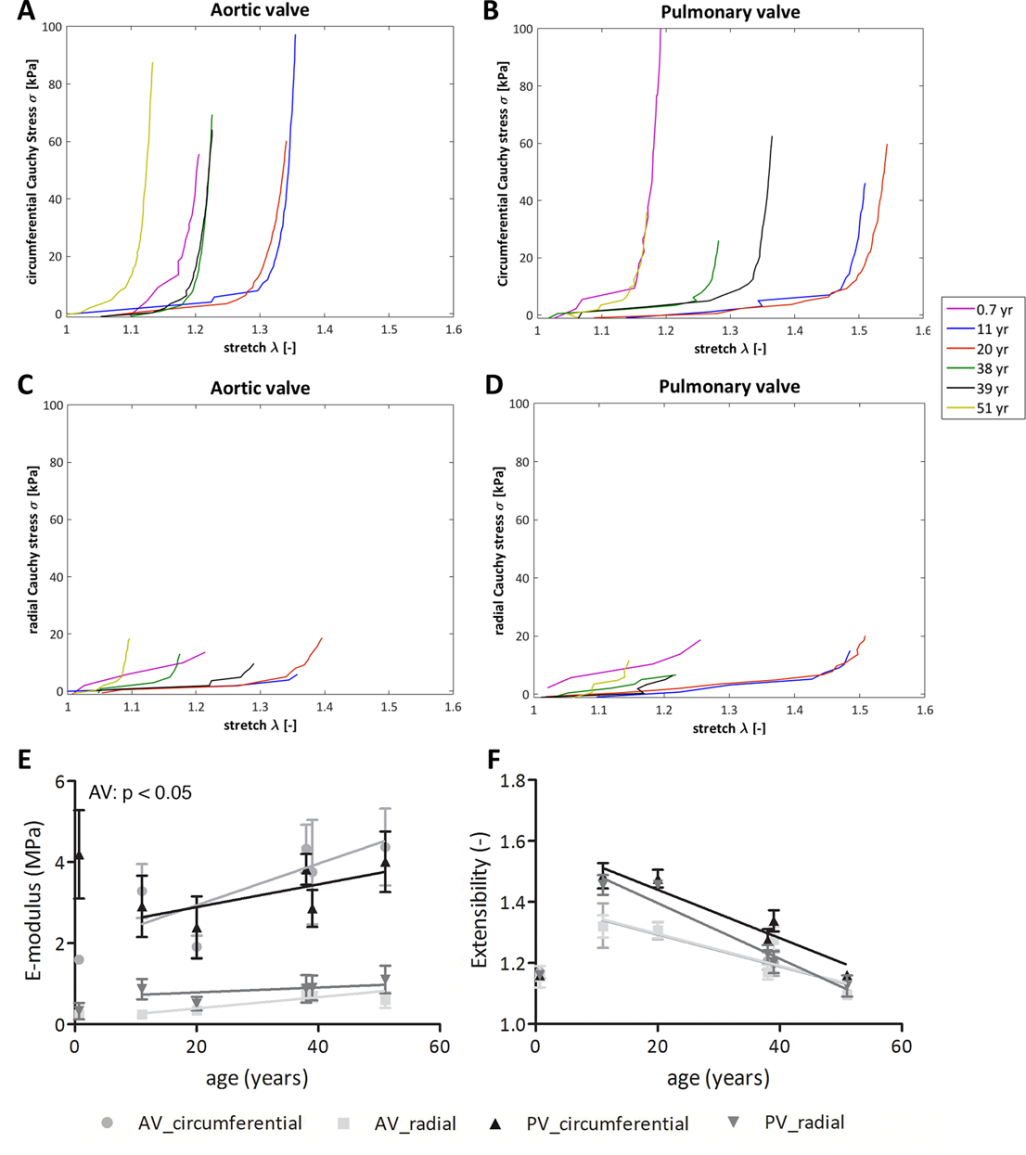
Averaged stress-strain curves, E-modulus at high strains, and extensibility obtained from biaxial tensile tests. Averaged stress-strain curves (A, B), E-modulus at high strains (C), and extensibility (D) obtained from biaxial tensile tests. The E-moduli (E) in both circumferential and radial direction increase with age in the aortic valve (p<0.05). In the pulmonary valve the E-modulus does not change with age. The leaflets of the 11 and 20-year-old donors are more extensible than the adult leaflets (F). In addition, in these young donors, the pulmonary leaflets were slightly more extensible than the aortic leaflets. As the data of the 8-month old donor could be considered as an outlier, we also performed linear regression analysis of the data set excluding the 8-month data and added the corresponding linear regression lines to Figs E and F. This reveals that after the age of 11, leaflet extensibility significantly decreases with age in circumferential (AV: p<0.01, PV: p<0.05) and radial direction (PV: p<0.01). AV: aortic valve, PV: pulmonary valve.

In the aortic valve leaflets, the low-strain stiffness of the belly (p<0.01), as well as the commissure (p<0.05) increased with age (Fig 6A and 6B). This increase was striking from adolescence (belly: 3.5 kPa; commissure: 7.0 kPa) to adulthood (belly: 10.5 kPa; commissure: 20.8 kPa). Biaxial tensile testing showed that aortic leaflet stiffness increased both in circumferential and radial direction (Fig 7A and 7B; p<0.05). In the pulmonary valve leaflets, the low-strain stiffness increased with age only in the commissures (Fig 6B; p<0.01), although the belly showed a 2-fold increase (p<0.05) in the low-strain stiffness from adolescence (4.5 kPa) to adulthood (10.3 kPa). Here, no age-related differences in circumferential and radial stiffness were observed. In both valves and at all ages, the stiffness in circumferential direction was higher than in radial direction, indicating anisotropic behavior.

The leaflets of the pediatric (11 year) and adolescent (20 year) valves were more extensible than the adult leaflets (Fig 7C and 7D) and also more extensible on the pulmonary side than on the aortic side. On the other hand, leaflet extensibility of the 8-month old valve was relatively low and comparable to the extensibility of the adult leaflets for both the aortic and pulmonary valve. Although this may reflect the natural variability amongst the donor valves, the 8-month valve could also be considered as an outlier in the absence of more valves of comparable age. Linear regression analysis excluding the data from the 8-month old valve revealed that leaflet extensibility in circumferential direction decreased significantly with age for both the aortic and pulmonary valves (p < 0.05, lines in 7E). For the pulmonary valve, also the extensibility in radial direction decreased significantly with age (p < 0.01). The E-moduli shows no correlation with age when excluding the data from the 8-month old valve (lines in 7F).

### Leaflet mechanical properties are related to matrix composition and maturation

In the aortic valve, the belly stiffness positively correlated with collagen content (p<0.05; r = 0.62). Thus, tissue stiffness increases with increasing collagen content. Furthermore, the stiffness in circumferential direction positively correlated with HP cross-link density (p<0.05; r = 0.9).

In the pulmonary valve, a negative correlation was observed between sGAG content and commissure stiffness (p<0.05; r = -0.56), meaning that the stiffness is decreasing with increasing sGAG content. In addition, positive correlations between HP and LP cross-link densities and belly stiffness were observed for this valve (p<0.01; r = 0.71 and p<0.05; r = 0.58 for the HP and LP cross-links, respectively).

### Reported valvular hemodynamics do not correlate with observed valve geometry and tissue properties in adults

**Fig 8:**
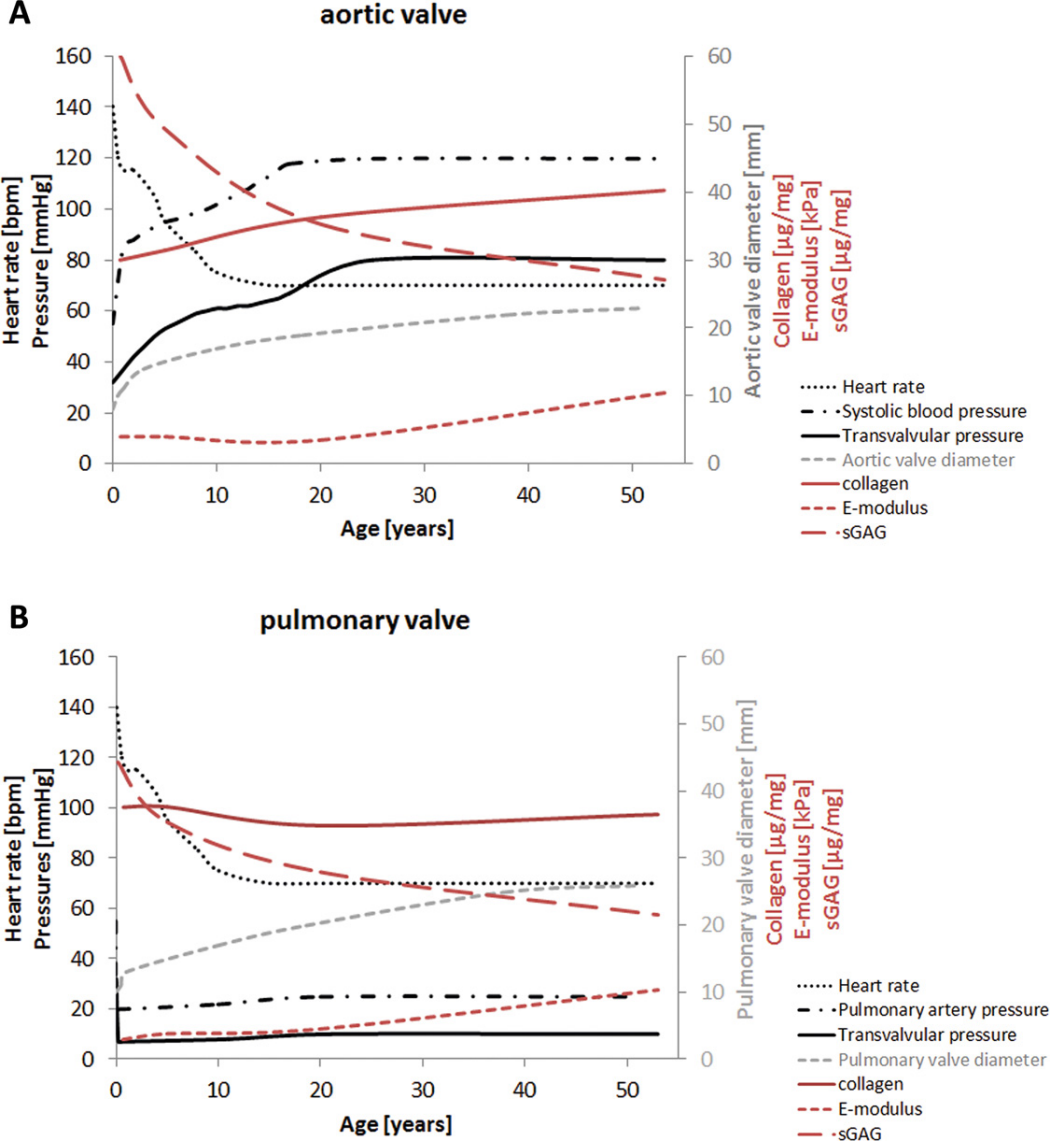
Valve hemodynamics during postnatal life in the aortic and pulmonary valve. Trend lines for reported data on valve hemodynamics (black) and our measured data on annulus diameter (grey), matrix composition, and stiffness of the belly (red) during postnatal life in the aortic (A) and pulmonary valve (B). Left-sided pressures increase during childhood, while the right-sided pressures decrease to adult values rapidly after birth. Hemodynamic data are collected from several databases, including The National Heart Lung and Blood Institute, MedScape and literature. Trend lines for our experimental data were obtained using the mean values of the data sets depicted in Figs 2, 4 and 6.

In an attempt to correlate valve composition, geometry, and mechanical properties to valve hemodynamic loading, age-specific results were compared with average data on valvular hemodynamics reported in literature (heart rate, pressures). While changes in matrix composition and valve dimensions observed in the young age groups seem to coincide with reported changes in hemodynamic conditions, ongoing changes in dimensions and matrix composition later in life are difficult to relate to the more stable average hemodynamic conditions and may therefore also reflect the consequences of ageing. For instance, gradual increases in collagen content and stiffness, as well as a decrease in sGAG content, are observed for both the aortic and pulmonary valves during adult age, while hemodynamic loading conditions can be considered more stable (Fig 8).

## Discussion

To determine structure-function properties, valve remodeling and differences thereof between pulmonary and aortic heart valves with age, we obtained data on valve dimensions, composition, maturation and mechanical properties of pairs of human aortic and pulmonary cryopreserved (donor) valve leaflets across a broad range of age. All studied leaflets, including the fetal leaflets, demonstrated a three-layered structure, with mainly collagen type I in the fibrosa, sGAG in the spongiosa, and elastin in the ventricularis. Aikawa *et al*. described that the three-layered structure, with elastin in the ventricularis, becomes apparent at 36 weeks of gestation [16], whereas our data demonstrated the presence of elastin already by week 21. This discrepancy might be caused by differences in staining techniques, as Votteler *et al*. demonstrated tropoelastin/elastin expression in the semilunar valve leaflets as early as week 7 of gestation and concluded that Verhoeff’s containing ECM-visualizing stains fail to identify developing, immature elastic fibers [24]. This corresponds with our findings that elastin was hardly observed in the fetal valves with the Verhoeff-Van Gieson staining, but clearly observed with the immunofluorescent straining.

With respect to valve dimensions, we found that the annulus increases rapidly during the first years of life, and slowly thereafter, even at older age. This can be due to growth or to a decrease in compliance with older age, causing dilatation of the annulus, as suggested by Merryman [25]. Annulus dilation may hinder movement and/or proper coaptation of the leaflets, thereby promoting regurgitation [26]. Interestingly, however, we also observed an increase in leaflet size with age. This may indicate an active remodeling or growth process to ensure proper valve closure. The thickness of the leaflets did not change with age. However, in the adolescent and adult age groups, the belly was thicker than the commissures. While this can be explained from the required load bearing capacity of the belly region [27,28], it might also be partly related to the presence of fibrotic spots, which were observed in the fibrosa of all adult and some adolescent leaflets.

From fetal to adolescent and adult age, the number of valvular interstitial cells decreased, which might be attributed to an increased cell proliferation-to-apoptosis ratio in young valves [16]. At older age, the number of cells remained constant and the cells mainly homed in the ventricularis and spongiosa. After birth, the cellular phenotype is assumed to be regulated by environmental cues [26], such as local tissue strains due to hemodynamic loading. Similar to the findings of Aikawa *et al*., the cells in the leaflets of the fetal and 2- and 8-month old donor were αSMA-positive, suggesting an activated myofibroblast phenotype relevant for fast matrix synthesis and remodeling [29,30]. Interestingly, no differences in αSMA-positive cells between the aortic and pulmonary valve were observed, suggesting that both valves have comparable remodeling capacity at young age. In the older aortic leaflets only quiescent cells (no αSMA) were observed, but mainly in the spongiosa and ventricularis and not in the fibrosa. This is surprising, since the fibrosa of all older valves showed evidence of fibrotic areas, suggesting ongoing matrix remodeling. Possibly this remodeling is so slow or passive due to ongoing collagen cross-linking, that it is not reflected by VIC presence or phenotype. Alternatively, valvular endothelial cells on the fibrosa side may contribute to the onset of fibrosis via shear stress-induced increase in inflammatory receptors [31] and decrease in fibrosis inhibitors [32]. In the absence of VICs, these negative effects cannot be suppressed [33] and will continue to contribute to valve degeneration.

Our mechanical characterization indicated anisotropic mechanical behavior in all studied age groups, similar to other studies using porcine or human valves from single age groups [6,13,15,34–37]. In addition to this, the adult valve leaflets were less extensible than the pediatric and adolescent leaflets. This was previously reported for porcine [6] and human [15] aortic leaflets and our study adds the pulmonary valve leaflets to that list. Leaflet stiffness was shown to increase with both increasing collagen content and decreasing sGAG content. Merryman hypothesized that ECM stiffness increases with age [25], which might be explained from our observation of the shift towards more collagen compared to sGAG in the ageing heart valve leaflets. The increase in stiffness with age can also be explained from the observed increase in collagen cross-links with age, which corresponds to previous work from our group [36].

The valvular hemodynamics are unchanging during adult life, while the geometry, composition, and mechanical properties of the valve do change. The change in annulus diameter and leaflet dimensions during adult age, being either the consequence of growth or dilation, seems to correspond with the decrease in extensibility of the leaflets. It might be argued that a loss of tissue extensibility is compensated by growth of the leaflets to ensure valve closure with age.

Remarkably, we hardly found differences in studied parameters between the aortic and pulmonary leaflets, except for the thickness of the leaflet belly. We even found comparable mechanical properties between the aortic and pulmonary leaflets, similar to others [14,35]. It is peculiar that the evolution of mechanical properties of both valve leaflets is similar, although the transvalvular pressures on both valves remain distinct after birth (Fig 8).

Our data can be used as input for designing TE heart valves or to assess the outcomes of (other) living prostheses with known properties. In our group, Mol *et al*. [38] and Kortsmit *et al*. [39,40] investigated matrix composition and mechanical properties of TE heart valves created from human myofibroblasts seeded into rapidly degrading scaffolds. Although it is difficult to compare values of native and engineered heart valves due to possible contributions of scaffold remnants in the TE heart valves, the DNA and sGAG content of engineered heart valves were similar, while the collagen content is much lower, compared to native valves. In the engineered valves there is more sGAG compared to collagen, while in the adult native heart valves there is more collagen compared to sGAG. Since the ECM composition of the heart valve is related to maturation and mechanical properties, it might be suggested that TE protocols should be optimized to improve the hydroxyproline content and therewith the (mechanical) functionality of these heart valves. TE valves are stiffer when compared to the mechanical properties found in the present study for native valves. This suggests that not only the matrix composition and maturation are important for defining the mechanical functionality, but especially the matrix architecture seems to be important for the mechanical functionality [41,42]. Thus, to increase the long-term *in vivo* functionality of the TE heart valves, not only the matrix composition and maturation should be optimized, also the collagen architecture that defines the anisotropic properties of the leaflets should be studied in native valve leaflets to improve HVTE.

In conclusion, we present here for the first time an complete overview of tissue mechanical properties, matrix composition and maturation of pairs of human aortic and pulmonary valve leaflets of different age groups (fetal, child, adolescent, adult). Interestingly, the only differences between the aortic and pulmonary valve were found to be in thickness and hemodynamic properties, while the differences in tissue composition and mechanical properties were minimal for all age groups. This suggests that future therapies can focus on one living replacement for both the pulmonary and the aortic valve. Further, this study proposes that future living valve replacements for children should have a remarkably different ECM composition (lower collagen-to-sGAG ratio) and architecture to result in a lower stiffness as compared to the adult replacements.

## Study Limitations

This study presents data on cryopreserved valves across a broad range of ages and includes sets of pulmonary and aortic valves from the same donors. As such it adds significantly to exiting studies on human aortic valves and porcine aortic valves. Still, numbers are relatively low and described trends and correlations are based on the sparsely available donor valves obtained during >5 year of research. Ideally, a large and normally distributed data set would be ideal and allow for regression analyses with age as a continuous variable to provide for sound conclusions on age-related effects, but this is unrealistic for donor valves. Therefore, we provided correlation graphs (Figs 2 and 4–7), as well as a more pragmatic distribution of age groups, based on the available but limited data set. As such, the child group included all *available* valves in children’s ages (2 months-11 years), the adolescent group the valves of donor 18–22 years of age and the adult group the other available valves (only above the age of 35). To strengthen our data we encourage other researchers to add to our data, which is available upon request.

Despite reported similarities in tissue microstructure and mechanical properties of cryopreserved valves [17, 19], it is difficult to assume the complete absence of cryopreservation effects on tissue biochemical composition and function. For instance, it has been reported that valvular GAG content is influenced by cryopreservation, but is better preserved when the valves are frozen in open leaflet position [43], as was the case in our study. Ongoing studies in our labs therefore compare fresh versus cryopreserved valves with respect to mechanical behavior and biochemical composition. Nevertheless, assuming comparable impact of cryopreservation on tissues of different age groups, all valves in the present study underwent similar freeze-thawing protocols, suggesting that the observed trends are likely to be attributed to age or age-related changes and not to cryopreservation.

The mechanical properties of the valve leaflets in the present study were obtained from experimental tensile and indentation studies only and presented as leaflet extensibility and (compressive and tensile) moduli. One should note that indentation and tension lead to different material behavior and therefore the compressive and tensile moduli cannot be directly related. For a complete description and prediction of mechanical behavior of the leaflets, however, a numerical-experimental approach comprising a constitutive model of the non-linear visco-elastic behavior is required. While this has been applied in recent studies by our group for both tensile [20] and indentation test [44] of the human native heart valve sets, here we chose a more comprehensive representation of leaflet properties, which is quite common in papers addressing in-vivo outcomes of engineered heart valves and heart valve pathology.

## Supporting Information

S1 Appendix Extended description of the cell phenotype, tissue composition and maturation analysis. (DOC)

S1 Fig Representative histological and immunofluorescent stainings on pulmonary valves. Figures A-E represent the whole pulmonary valve (leaflet, hinge region, and wall, while in E-W a representative part of the leaflet is shown. (A, C-F, H-J) Verhoeff-Van Gieson staining for collagen (red) and elastin (black). (B, G) Elastin was observed in the fetal valve using immunofluorescence (red). (K-N) Collagen type I (red) was predominant in the fibrosa. (O-R) Safranin-O staining showed proteoglycan presence (red/orange) mainly in the spongiosa and the hinge region. (S-W) αSMA (immunofluorescence; green) with cell nuclei (in blue). Scale bar: 500 μm. l: leaflet; w: wall; f: fibrosa; s: spongiosa; v: ventricularis.
(TIF)

S1 Table Age groups composition and experiments. Overview of the composition of the age groups and the experiments performed on the sets of pulmonary and aortic heart valve leaflets (indicated with an ‘X’).
(DOCX)
