# Supplementary material for: Age-Dependent Changes in Geometry, Tissue Composition and Mechanical Properties of Fetal to Adult Cryopreserved Human Heart Valves
Source: PLoS One. 2016 Feb 11;11(2):e0149020. doi: 10.1371/journal.pone.0149020 (PMC4750936; doi:10.1371/journal.pone.0149020)
Supplement: S1 Appendix — (DOC) [file pone.0149020.s001.doc]

# Appendix S1. Extended description of the cell phenotype, tissue composition and maturation analysis.

Qualitative analyses

The sections were studied by hematoxylin and eosin (H&E) staining for general tissue composition, Masson Trichrome (MTC kit, Sigma HT15) for collagen, Verhoeff-Van Gieson staining for collagen and elastin, and Safranin-O staining for proteoglycans. Additionally, matrix components and cellular phenotype were assessed with immunofluorescent stainings. After pretreatment with 6 M guanidine-HCl, 50 mM dithiothreitol, 20 mM Tris (pH 8.0) and washing with 20 mM Tris (pH 8.0) for antigen retrieval, elastin was analyzed with a polyclonal rabbit IgG antibody against elastin (abcam, 1:500). Collagen type I and type III antigens were retrieved by boiling in 10 mM Sodium citrate-HCl buffer (pH 6.0) and incubation in 0.04% pepsin buffer, respectively. Thereafter, collagen type I and III were determined with a polyclonal rabbit IgG antibody against collagen type I (abcam, 1:250) and with polyclonal rabbit IgG antibody against collagen type III (abcam, 1:200). The phenotypic marker alpha smooth muscle actin (αSMA) antigen was retrieved by boiling in 10 mM sodium citrate-HCl buffer (pH 6.0) and analyzed with a monoclonal IgG2a mouse anti-human antibody against αSMA (Sigma, 1:500). αSMA was visualized with a goat anti-mouse IgG2a Alexa 488 (Molecular Probes, 1:300 dilution), while the matrix components were visualized with donkey anti-rabbit Alexa 555 (Molecular Probes, 1:300 dilution). Additionally, cell nuclei were stained with 4’,6-diamidino-2-phenylindole (DAPI).

Quantitative analyses

The amount of DNA (representing total cell number) in the leaflets was quantified using the Hoechst dye method [1] with a reference curve prepared of calf thymus DNA (Sigma). A modification of the assay described by Farndale *et al.* (1986) [2] with shark cartilage chondroitin sulfate as a reference was used to measure the content of sGAG. To determine the hydroxyproline quantity, as a measure for the collagen content, the assay according to Huszar *et al.* (1980) [3] and a reference of trans-4-hydroxyproline (Sigma) was used. The content of DNA, sGAG and hydroxyproline was expressed as μg per mg dry weight. The number of mature collagen hydroxylysyl pyridinoline (HP) and lysyl pyridinoline (LP) cross-links, as a measure of tissue maturity, was measured in the digested samples using high-performance liquid chromatography as described previously [4-6]. The number of HP and LP cross-links was expressed per collagen triple helix, thus representing cross-link density.

## References

1. Cesarone CF, Bolognesi C, Santi L. Improved microfluorometric DNA determination in biological material using 33258 Hoechst. *Analytical biochemistry*. 1979;100(1):188–97.

2. Farndale RW, Buttle DJ, Barrett a J. Improved quantitation and discrimination of sulphated glycosaminoglycans by use of dimethylmethylene blue. *Biochimica et biophysica acta*. 1986;883(2):173–7.

3. Huszar G, Maiocco J, Naftolin F. Monitoring of collagen and collagen fragments in chromatography of protein mixtures. *Analytical biochemistry*. 1980;105(2):424–9.

4. Bank RA, Jansen EJ, Beekman B, te Koppele JM. Amino acid analysis by reverse-phase high-performance liquid chromatography: improved derivatization and detection conditions with 9-fluorenylmethyl chloroformate. *Analytical biochemistry*. 1996;240(2):167–76.

5. Robins SP, Duncan A, Wilson N, Evans BJ. Standardization of pyridinium crosslinks, pyridinoline and deoxypyridinoline, for use as biochemical markers of collagen degradation. *Clinical chemistry*. 1996;42(10):1621–6.

6. Bank RA, Beekman B, Verzijl N, de Roos JA, Sakkee AN, TeKoppele JM. Sensitive fluorimetric quantitation of pyridinium and pentosidine crosslinks in biological samples in a single high-performance liquid chromatographic run. *Journal of chromatography. B, Biomedical sciences and applications*. 1997;703(1-2):37–44.
