# Supplementary figures and images for: Age-Dependent Changes in Geometry, Tissue Composition and Mechanical Properties of Fetal to Adult Cryopreserved Human Heart Valves
Source: PLoS One. 2016 Feb 11;11(2):e0149020. doi: 10.1371/journal.pone.0149020 (PMC4750936; doi:10.1371/journal.pone.0149020)

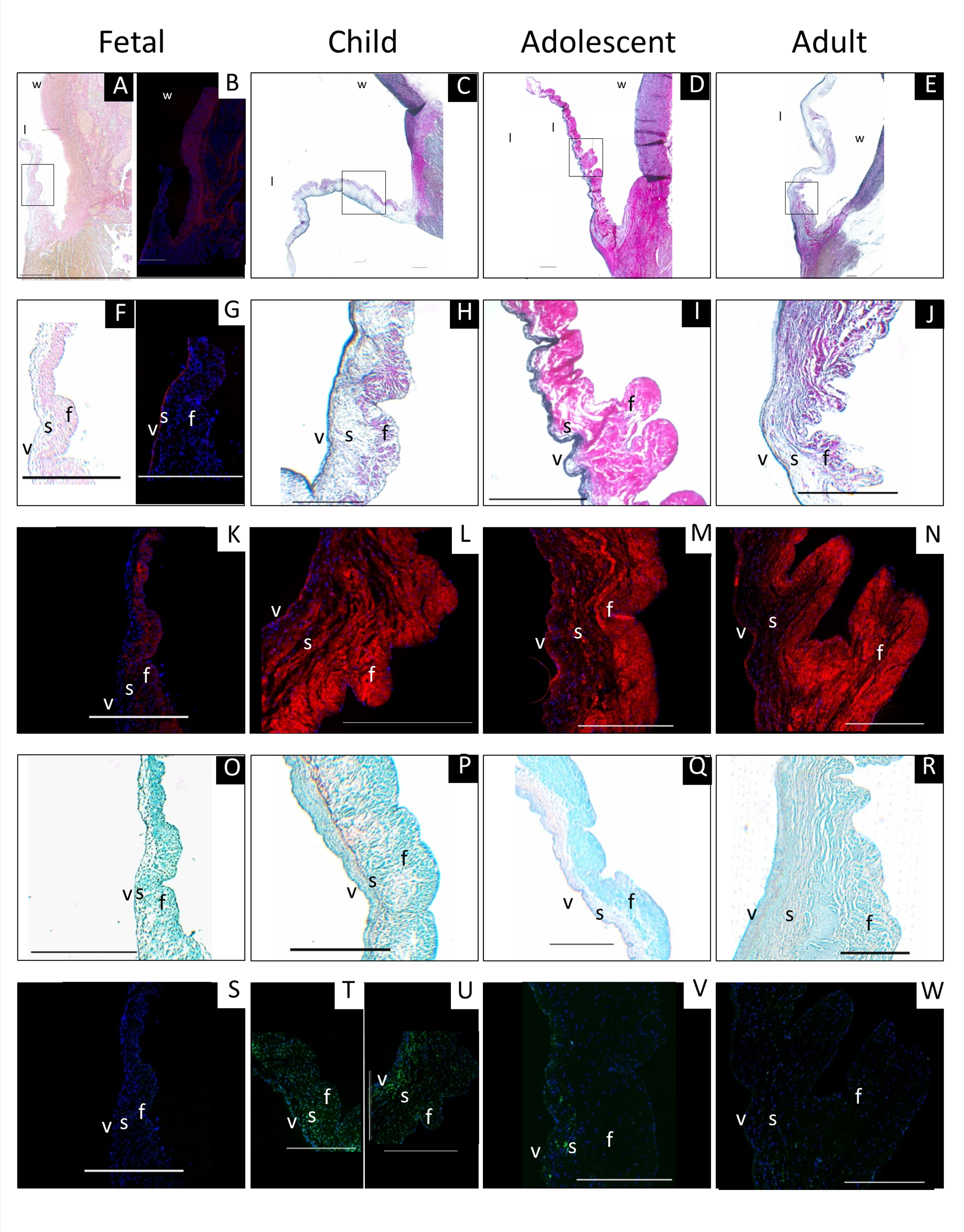

Supplement: S1 Fig — Figures A-E represent the whole pulmonary valve (leaflet, hinge region, and wall, while in E-W a representative part of the leaflet is shown. (A, C-F, H-J) Verhoeff-Van Gieson staining for collagen (red) and elastin (black). (B, G) Elastin was observed in the fetal valve using immunofluorescence (red). (K-N) Collagen type I (red) was predominant in the fibrosa. (O-R) Safranin-O staining showed proteoglycan presence (red/orange) mainly in the spongiosa and the hinge region. (S-W) αSMA (immunofluorescence; green) with cell nuclei (in blue). Scale bar: 500 μm. l: leaflet; w: wall; f: fibrosa; s: spongiosa; v: ventricularis. (TIF) [file pone.0149020.s002.tif]
