# Supplementary material for: Age-Dependent Changes in Geometry, Tissue Composition and Mechanical Properties of Fetal to Adult Cryopreserved Human Heart Valves
Source: PLoS One. 2016 Feb 11;11(2):e0149020. doi: 10.1371/journal.pone.0149020 (PMC4750936; doi:10.1371/journal.pone.0149020)
Supplement: S1 Table — Overview of the composition of the age groups and the experiments performed on the sets of pulmonary and aortic heart valve leaflets (indicated with an ‘X’). (DOCX) [file pone.0149020.s003.docx]

**Table S1. Age groups composition and experiments.**

Overview of the composition of the age groups and the experiments performed on the sets of pulmonary and aortic heart valve leaflets (indicated with an ‘X’).

| **Group** | **Sex** | **Cause of death** | **Age** | **histology** | **Biochemical assays** | **Cross-links** | **Indentation tests** | **Biaxial tensile tests** |
| --- | --- | --- | --- | --- | --- | --- | --- | --- |
| fetal | - | Abortion | 21 weeks | X |  |  |  |  |
| Child | Male | Subaranchoid bleeding | 2 months | X | X |  | X |  |
|  | Female | Suffocation | 8 months | X | X | X | X | X |
|  | Male | Post-anoxic encephalopathy | 2.2 year | X | X |  | X |  |
|  | Male | Respiratory insufficiency | 5 year | X | X | X | X |  |
|  | Male | Suffocation | 11.2 year | X | X | X | X | X |
| Adolescent | Female | Trauma (SHT) | 18.2 year | X | X | X | X |  |
|  | Female | Pulmonary embolus | 18.7 year | X | X | X | X |  |
|  | Male | Bleeding / Hypovolemic shock | 20.3 year | X | X | X | X | X |
|  | Female | Trauma (SHT) | 22.5 year | X | X | X | X |  |
| Adult | Male | Trauma (SHT) | 38.5 year | X | X | X | X | X |
|  | Female | Suicide | 39.9 year | X | X | X | X | X |
|  | Male | Trauma (SHT) | 43.9 year | X | X | X | X |  |
|  | Male | Cerebrovascular accident | 48.3 year | X | X | X | X |  |
|  | Male | Cerebrovascular accident | 51.4 year | X | X | X | X | X |
|  | Male | Myocardial Infarction | 53.4 year | X | X | X | X |  |
